# The importance of modelling the spread of insecticide resistance in a heterogeneous environment: the example of adding synergists to bed nets

**DOI:** 10.1186/1475-2875-11-258

**Published:** 2012-08-02

**Authors:** Susana Barbosa, Ian M Hastings

**Affiliations:** 1 Liverpool School of Tropical Medicine, Pembroke Place, Liverpool, UK

## Abstract

**Background:**

Insecticides are an effective and practical tool for reducing malaria transmission but the development of resistance to the insecticides can potentially compromise controls efforts. In this study a mathematical model was developed to explore the effects on mosquito populations of spatial heterogeneous deployment of insecticides. This model was used to identify important parameters in the evolution of insecticide resistance and to examine the contribution of new generation long-lasting insecticidal bed nets, that incorporate a chemical synergist on the roof panel, in delaying insecticide resistance.

**Methods:**

A genetic model was developed to predict changes in mosquito fitness and resistance allele frequency. Parameters describing insecticide selection, fitness cost and the additional use of synergist were incorporated. Uncertainty and sensitivity analysis were performed followed by investigation of the evolution of resistance under scenarios of fully effective or ineffective synergists.

**Results:**

The spread of resistance was most sensitive to selection coefficients, fitness cost and dominance coefficients while mean fitness was most affected by baseline fitness levels. Using a synergist delayed the spread of resistance but could, in specific circumstances that were thoroughly investigated, actually increase the rate of spread. Different spread dynamics were observed, with simulations leading to fixation, loss and most interestingly, equilibrium (without explicit overdominance) of the resistance allele.

**Conclusions:**

This strategy has the potential to delay the spread of resistance but note that in an heterogeneous environment it can also lead to the opposite effect, i.e., increasing the rate of spread. This clearly emphasizes that selection pressure acting inside the house cannot be treated in isolation but must be placed in context of overall insecticide use in an heterogeneous environment.

## Background

Malaria is one of the most important parasitic infection in humans. Several initiatives from the international health community in the past decade have lead to an estimated drop in malaria associated mortality from around 1 million in 2000 to about 655,000 in 2010 according to the WHO [1], although an independent recent study reported the decrease to be from 1.82 million in 2004 to 1.24 million in 2010 [2].

Among the current recommended interventions to control the disease is the use of insecticidal nets or indoor residual spraying with insecticide to control vector mosquitoes populations [1]. A major issue arising from the intense deployment of insecticides is the development of resistance to the chemical agents [3]. It is a ubiquitous problem, regarded as a major hindrance in the control of malaria. Furthermore, the use of insecticides is not restricted to public health, in fact, around 90% of all insecticide is deployed in agriculture [4]. This potential spatial heterogeneity of insecticide deployment can give rise to a mixed environment for mosquito populations.

In the past mathematical models have been used to inform resistance management practices [5], determining the impact of different mosquito control intervention strategies including the protection conferred by bed nets [6], and, recently, to develop new approaches such as the idea of evolution-proof insecticides [7-9]. However, few [10] have considered the spread of resistance in a variable selection pressure context. Consequently a model is presented here that considers different niches in an environment, that can offer some insights on the importance of different parameters and their interactions in the dynamics of insecticide resistance. This approach is particularly suitable for investigating the impact of a specific long-lasting insecticidal net that is being developed. Vestergaard Frandsen has submitted a bed net prototype (PermaNet 3.0) for formal evaluation to the WHO Pesticide Evaluation Scheme that incorporates insecticide plus synergist.

Synergists are natural or synthetic chemicals, which increase the lethality and effectiveness of currently available insecticides, but that are nontoxic to insects on their own. They block the metabolic systems that would otherwise break down insecticide molecules, helping to restore chemical susceptibility that would require higher levels of the insecticide [11]. For this reason they are proposed for use in overcoming metabolic resistance and also to delay the manifestation and/or spread of resistance [12].

ln this bed net, synergist (Piperonyl butoxide - PBO) together with the pyrethroid deltamethrin are incorporated into the fibres on the roof panel of the net, while incorporating only deltamethrin on a lower dosage in the side panels. The rationale behind this approach approximates the ”two-in-one” concept for bed nets. Treating different parts of bed nets with different insecticides (e.g. combining pyrethroid insecticide, applied to the side panels of the bed net, together with carbamate insecticide on the roof [13]) has been suggested to confer advantages over the use of insecticides alone [14]. The assumption is that foraging female mosquitoes explore an occupied bed net from the top downwards (as the warm air and carbon dioxide that emanate from the sleeper move upwards ), i.e., will land on the roof first and make their way down the side panels. Restricting the synergist to the roof also allows the sides of the net to be made of a softer and more comfortable fiber for the user [15].

In this paper a general and flexible model was developed by expanding the usual genetic models to account for spatial and sexual heterogeneities in insecticides exposures. Statistical tools as partial rank correlation coefficients, logistic regression and classification trees were used to explore specific situations of synergist application and to uncover the dynamics of resistance.

## Methods

### Model

A population genetic model was designed that predicts changes in mean fitness and resistant allele frequency as outcome variables to explore the relative contribution of each different environmental niche to the dynamics of the population insecticide resistance status. Mean fitness assesses the potential effectiveness of control strategies at decreasing the population while change of allele frequency between generations quantifies selection pressure for resistance.

The model is deterministic, i.e., based on the approximation of an infinitely large population size so that stochastic fluctuations of allele frequencies can be neglected. Investigations of the changes in allele frequency caused by natural selection are based upon the assumption that selection operates through differential survival of the zygote from birth to maturity. It assumes that random mating occurs among all adults pooled across all niches, and that progeny are then randomly distributed among the niches. Resistance is determined by one allele at one locus [16-18](S: insecticide susceptible allele; R: insecticide resistance allele).

Table 1 defines the fitness of each genotype for each different niches. It also defines the proportions exposed to each niche, that sum to 1, which implies that a mosquito can only encounter a single niche in a generation.

**Table 1:** Model structure: niches, exposure and genotype fitnesses within each niche

		**Niches**		
	**Insecticide free**	**Non public**	**ITN**	**ITN + Synergist**
Exposure males	1−(*α*_*mo*_ + *α*_*mi*_)	*α*_*mo*_	*α*_*mi*_(1−*β*_*m*_)	*α*_*mi*_*β*_*m*_
Exposure females	1-(*α*_*fo*_ + *α*_*fi*_)	*α*_*fo*_	*α*_*fi*_ (1-*β*_*f*_)	*α*_*fi*_*β*_*f*_
Fitness SS	1	1−*φ*_*o*_	1−*φ*_*i*_	(1−*φ*_*i*_)*k*
Fitness RS	1−*h*_*n*_*z*	(1−*φ*_*o*_) + *h*_*o*_*s*_*o*_	(1−*φ*_*i*_) + *h*_*i*_*s*_*i*_	[(1−*φ*_*i*_) + *h*_*i*_*s*_*i*_]*k*
Fitness RR	1-*z*	(1−*φ*_*o*_) + *s*_*o*_	(1−*φ*_*i*_) + *s*_*i*_	[(1−*φ*_*i*_) + *s*_*i*_]*k*

Four niches were considered: 

1- Insecticide free (n): it can be an area either inside or outside a household;

2- Non public-health related insecticide deployment (o): typically insecticide use in agriculture and households. These are deployed outwith of public health mosquito control campaigns, and generally out of the control of public health officials; The subscript ‘o’ is used for brevity, noting that casual use inside the house, e.g. mosquito coils, would also be included in this class;

3- Insecticide-treated bed nets (ITN);

4- Insecticide-treated bed nets with synergist on the top of the net (ITN + Synergist);

There is likely to be differential exposure to insecticide and hence different selection pressure in the sexes, since only females feed on humans and are, therefore, the ones most likely to enter human habitations and encounter insecticides. Consequently, the proportion of mosquitoes (*α*) that encounter each niche was differentiated by sexes and genotype fitness was calculated separately. Fitness of the genotype SS in the insecticide free niche was considered as the reference fitness level, and other genotypic fitnesses were measured relative to this fitness, which was taken to be 1.

Different selection (*s*) and dominance (*h*) parameters were defined for each niche, except for the insecticide free. There is by definition no insecticide exposure in the insecticide free niche, so *s* is replaced by *z* (the cost of carrying a resistance allele). Dominance is not an intrinsic property of the alleles, it depends on the environment in which they are expressed, thus the differences in dominance coefficients between niches. High levels of insecticide may render the resistance allele recessive, because only homozygotes survive, while low levels may allow survival of both heterozygotes and resistant homozygotes rendering the allele dominant [19-21].

This is a highly flexible genetic model, that includes a baseline fitness level *φ* for niches where insecticide is deployed, that captures the variable effects on fitness of being fully susceptible to insecticides [22]. For example, setting *φ*_*o *_=* φ*_*i*_= 1 means SS genotype always killed when meeting insecticides while setting *φ*_*o *_= 0.9 means 10% of SS will survive exposure in the non public niche. It also allows the fitness of a resistance homozygote meeting a ITN to be less than 1 and therefore smaller than a susceptible homozygote in a insecticide free niche, reflecting the fact that a fully resistance genotype may not be completely impervious to the insecticide. For example setting *φ*_*o *_= 0.9,*h*_*o *_= 0.2,*s*_*o *_= 0.6 means that 10% of SS genotypes, 22% of RS and 70% of RR survive exposure in the non public-health related insecticide deployment niche.

Two parameters were included that relate to the additional use of synergist: *k* that quantifies synergist efficiency and *β*the proportion of mosquitos meeting both insecticide and synergist in the bed net. It is assumed that synergist exposure is equally efficient across genotypes. For example, if the probability of surviving bed net contact for *SS*, *RS* and *RR* genotypes is 10%, 22%, 70% (see above) and *k *= 0.1, the proportion surviving bed net plus synergist falls to 1%, 22% and 7%, respectively. It would be straightforward to include separate *k* values for each genotype if the synergist impact differed between genotypes. Description of all parameters on Table 2.

**Table 2:** Parameters, symbols and subscripts used in the construction of the model

**Parameter**	**Symbol**
Dominance coefficient in each niche	*h*
Fitness cost of carrying a resistance allele	*z*
Selection coefficient in each niche	*s*
Baseline fitness level of susceptible homozygote	
in niches were insecticide is deployed	*φ*
Proportion of mosquitoes encountering a	
particular niche	*α*
Proportion of mosquitoes encountering ITN	
that also encounter the synergist	*β*
Impact of synergist	*k*
*k*=0; synergist completely effective: all mosquitoes	
encountering insecticide plus synergist die	
*k*=1; synergist completely ineffective: mosquitoes	
encountering insecticide plus synergist die	
at the same rate as those encountering insecticide alone	
(the model tracks survival in different niches	
-Table 1, so the impact is on a reverse scale)	
Subscripts	
Male	*m*
Female	*f*
Insecticide free	*n*
Deployment of insecticide outside the house	*o*
Deployment of insecticide inside the house	*i*

Based on the model described on Table 1 the fitness (*W * with appropriate subscripts) across all niches of each genotype will be [23]:

Males,

(1) $$\begin{array}{l} W_{m,\text{ss}}=1-(\alpha_{\text{mo}}+\alpha_{\text{mi}})+\alpha_{\text{mo}}\;(1-\varphi_{o})\\ \;\;\;+\alpha_{\text{mi}}\;(1-\beta_{m})\;(1-\varphi_{i})\\ \;\;\;+\alpha_{\text{mi}}\;\beta_{m}\;(1-\varphi_{i})\;k \end{array}$$

(2) $$\begin{array}{l} W_{m,\text{rs}}=[1-(\alpha_{\text{mo}}+\alpha_{\text{mi}})]\;(1-h_{n}\;z)\\ \;\;\;+\alpha_{\text{mo}}[(1-\varphi_{o})+h_{o}\;s_{o}]\\ \;\;\;+\alpha_{\text{mi}}\;(1-\beta_{m})\;[(1-\varphi_{i})+h_{i}\;s_{i}]\\ \;\;\;+\alpha_{\text{mi}}\;\beta_{m}\;\;[\;(1-\varphi_{i})+h_{i}\;s_{i}\;]\;k \end{array}$$

(3) $$\begin{array}{l} W_{m,\text{rr}}=[1-(\alpha_{\text{mo}}+\alpha_{\text{mi}})]\;(1-z)\\ \;\;\;+\alpha_{\text{mo}}[(1-\varphi_{o})+s_{o}]\\ \;\;\;+\alpha_{\text{mi}}\;(1-\beta_{m})\;[(1-\varphi_{i})+s_{i}]\\ \;\;\;+\alpha_{\text{mi}}\;\beta_{m}\;\;[\;(1-\varphi_{i})+s_{i}\;]\;k \end{array}$$

Females,

(4) $$\begin{array}{l} W_{f,\text{ss}}=1-(\alpha_{\text{fo}}+\alpha_{\text{fi}})+\alpha_{\text{fo}}\;(1-\varphi_{o})\\ \;\;\;+\alpha_{\text{fi}}\;(1-\beta_{f})\;(1-\varphi_{i})+\alpha_{\text{fi}}\;\beta_{f}\;(1-\varphi_{i})\;k \end{array}$$

(5) $$\begin{array}{l} W_{f,\text{rs}}=[1-(\alpha_{\text{fo}}+\alpha_{\text{fi}})]\;(1-h_{n}\;z)\\ \;\;\;+\alpha_{\text{fo}}[(1-\varphi_{o})+h_{o}\;s_{o}]\\ \;\;\;+\alpha_{\text{fi}}\;(1-\beta_{f})\;[(1-\varphi_{i})+h_{i}\;s_{i}]\\ \;\;\;+\alpha_{\text{fi}}\;\beta_{f}\;\;[\;(1-\varphi_{i})+h_{i}\;s_{i}\;]\;k \end{array}$$

(6) $$\begin{array}{l} W_{f,\text{rr}}=[1-(\alpha_{\text{fo}}+\alpha_{\text{fi}})]\;(1-z)+\alpha_{\text{fo}}[(1-\varphi_{o})+s_{o}]\\ \;\;\;+\alpha_{\text{fi}}\;(1-\beta_{f})\;[(1-\varphi_{i})+s_{i}]\\ \;\;\;+\alpha_{\text{fi}}\;\beta_{f}\;\;[\;(1-\varphi_{i})+s_{i}\;]\;k \end{array}$$

If resistance allele frequency is *p*_*m*_/*p*_*f *_ and frequency of susceptible allele is *q*_*m*_/*q*_*f *_, after selection the genotypic frequencies will be:

(7) $$\begin{array}{l} RR_{m}=\frac{W_{m,\text{rr}}\;p_{m}\;p_{f}}{{\bar{W}}_{m}}\\ RS_{m}=\frac{W_{m,\text{rs}}\;(p_{m}\;q_{f}\;+\;p_{f}\;q_{m})}{{\bar{W}}_{m}}\\ SS_{m}=\frac{W_{m,\text{ss}}\;q_{m}\;q_{f}}{{\bar{W}}_{m}}\\ RR_{f}=\frac{W_{f,\text{rr}}\;p_{m}\;p_{f}}{{\bar{W}}_{f}}\\ RS_{f}=\frac{W_{f,\text{rs}}\;(p_{m}\;q_{f}\;+\;p_{f}\;q_{m})}{{\bar{W}}_{f}}\\ SS_{f}=\frac{W_{f,\text{ss}}\;q_{m}\;q_{f}}{{\bar{W}}_{f}} \end{array}$$

Where $\bar{W}$ are the mean fitness, given as the sum of the numerators:

(8) $$\begin{array}{l} {\bar{W}}_{m}=W_{m,\text{rr}}\;p_{m}\;p_{f}+W_{m,\text{rs}}\;(p_{m}\;q_{f}\;+\;p_{f}\;q_{m})\\ \;\;+W_{m,\text{ss}}\;q_{m}\;q_{f} \end{array}$$

(9) $$\;{\bar{W}}_{f}=W_{f,\text{rr}}\;p_{m}\;p_{f}+W_{f,\text{rs}}\;(p_{m}\;q_{f}\;+\;p_{f}\;q_{m})+W_{f,\text{ss}}\;q_{m}\;q_{f}$$

The frequency of the resistance allele in males after selection, i.e., in the mating pool for the next generation (*t* + 1), is

(10) $$p_{m,t+1}=\frac{W_{m,\text{rr}}\;p_{m}\;p_{f}+0.5\;W_{m,\text{rs}}\;(p_{m}\;q_{f}+p_{f}\;q_{m})}{{\bar{W}}_{m}}$$

and the corresponding frequency in females following selection is

(11) $$p_{f,t+1}=\frac{W_{f,\text{rr}}\;p_{m}\;p_{f}+0.5\;W_{f,\text{rs}}\;(p_{m}\;q_{f}+p_{f}\;q_{m})}{{\bar{W}}_{f}}$$

Under this model, the ratio of change of the gene frequency per generation is given by

(12) $$\Delta \;p_{m}=\frac{p_{m,t+1}}{p_{m,t}}$$

(13) $$\Delta \;p_{f}=\frac{p_{f,t+1}}{p_{f,t}}$$

All simulations started assuming Hardy-Weinberg equilibrium (HWE), but genotypes will move away from HWE due to differential selection on the sexes. This reflects their different degrees of exposure to different environments so that the resistance allele frequency will diverge slightly in the breeding individuals of each sex and their progeny genotypes will no longer be in HWE. Consequently, to allow for redistribution of resistance between the genotypes the chosen census point was at generations 10-11, based solely on intuition.

### Parameter values

The subjective part of the analysis lies in identifying plausible values and distributions for the parameters in Table 2.

Initial resistance allele frequency value, *p*_0_ = 0.001, was used in all calculations and was selected to reflect the initial stages of insecticide resistance (where most of the individual are expected to be heterozygotes). There is little field information available for the parameter values appropriate for *Anopheles gambiae* complex and parameter values vary depending on the species and the local environment. The range of values and distributions chosen were very broad to investigate general properties of the system; narrow distributions can be used to investigate specific situations.

The proportion of mosquitoes subject to a particular niche (*α*) were randomly selected from a uniform distribution but subjected to the constraint that the sum over all niches by sex is 1; values were randomly selected from the uniform distribution and then divided by the overall sum.

The proportion of males that meet the ITN, *α*_*mi *_was constrained to always be smaller than the proportion of females *α*_*fi*_ and smaller than 0.2 (less than 20% of the males of the population enter the household and contact the bed net) to reflect the belief that only a small proportion of males enter a household since they do not seek to blood feed on humans. The proportion of males that is expected to contact the top of the bed net, and be exposed to both insecticide and synergist is assumed to be very small, so we restricted the maximum value of *β*_*m*_ to 0.2.

### Uncertainty and sensitivity analysis

Simulations to understand the influence of each parameter on the outcome variables (mean fitness and change in resistance allele frequency) were performed using latin hypercube sampling (LHS) to generate a data set and partial rank correlation coefficients (PRCC) calculated to provide a quantitative measure of the impact of each parameter [24]. LHS techniques were first developed to explore the behavior of complex models in economics, engineering, chemistry and physics and have been used in models predicting the impact of insecticide-treated nets on malaria transmission [25].

The analysis was performed using R software [26] and implementation of LHS using package *lhs*. It does not allow for the specification of each variable distribution beforehand, so sampling was performed assuming a uniform distribution. Once the sample was generated, the uniform sample from a column (variable) could be transformed to the required distribution (Table 3) by using quantile functions (using the *qtriangle* comand in R).

**Table 3:** Parameters range of values used in simulations

	**Range of values**			
**Parameter**	**Minimum**	**Peak**	**Maximum**	**Distribution**
*p*_*m *_=* p*_*f*_	0.001			Constant
*h*_*n*_	0	0.5	1	Triangular
*h*_*o*_	0	0.5	1	Triangular
*h*_*i*_	0	0.5	1	Triangular
*z*	0	0.5	1	Triangular
*s*_*o*_	0	0.5	1	Triangular
*s*_*i*_	0	0.5	1	Triangular
*φ*_*o*_	0		1	Uniform
*φ*_*I*_	0		1	Uniform
*β*_*f*_	0		1	Uniform
*β*_*m*_	0		0.2	Uniform
*α*_*f*_^∗^	0	0.5	1	Triangular
*α*_*m*_^∗^	0	0.5	1	Triangular
*α*_*mi*_	0		0.2	Uniform
*k*	0		1	Uniform

^∗^Females: all female niches; Males: all male niches expect ITN/ITN+synergist.

A data set of 3,000 replications was generated, with random parameter sets and the corresponding values of the outcome variables using equations 8912 and 13. Ten replicates of this procedure were performed as suggested in [24] to investigate the predictive precision of model using LHS as the sampling method. This was achieved by analysing each replicate separately and verifying that results were consistent across ten replicates.

### Allele frequency ratio under two extreme scenarios of synergist effectiveness

Following uncertainty and sensitivity analysis the evolution of the frequency of the resistance allele was investigated. This was achieved by simulating a scenario with a fully effective synergist (*k *= 0), and the other extreme, a scenario where encountering the synergist had absolutely no effect (*k *= 1). The dataset consisted of 3,000 individual simulations that were run drawing values from Table 3 for the parameters. Each simulation was run twice, once with *k *= 0 and another one with *k *= 1. The ratio between the resistance allele frequency in the population in both scenarios at generation 10 ( $y=\frac{p_{10}|k=0}{p_{10}|k=1}$) indicates how fully effective synergists increase (*y *> 1) or decrease (*y *< 1) the spread of resistance.

Results included a counter-intuitive outcome that the inclusion of a synergist could lead to an increase in the rate of the spread of resistance (i.e. *y *> 1). Further investigation of this result was pursed by performing a logistic regression with a binary dependent variable (1 if *y *> 1 and 0 if *y *< 1), therefore quantifying how changes in the parameters values affect the odds of getting the unexpected outcome *y *> 1. In this regression only 14 parameters out of the 16 could be included. The parameters *α* are codependent, because they must sum to unity, so *α*_*mo*_, *α*_*fo*_ were excluded from the regression since they achieve the smaller PRCC values (see later).

### Classification trees

The model has a substantial number of parameters, 16, so the logistic regression becomes inefficient when considering all possible interactions between them. An alternative approach to logistic regression is classification trees, that sub-divide the parameter space into smaller regions, where the interactions are more manageable.

Classification trees are used to predict membership of cases in the classes of a categorical dependent variable (1 if *y *> 1 or 0 if *y *< 1) from their input parameters and were implemented using an algorithm that grows a binary tree [27]. At each internal node in the tree, a test is applied to the input parameters to identify the binary distinction which gives the most information about the class membership. The process is repeated at each resulting node, continuing the recursion until some stopping criterion is reached where it makes a prediction [27]. The threshold of complexity parameter (cp) was one of the stopping criteria used here, it ensures that any split that does not decrease the overall lack of fit by a factor of cp is not attempted; it can be preset or estimated using cross-validation. Here it was used cross-validation, which is a method for validating a procedure for model building, without an independent validation dataset. It includes any given random divisions of the data into 90% learning and 10% test sets [28]. The optimally sized tree was obtained by running 10-fold cross-validations on the data and by including another stopping criterion, a minimum of 50 observations in a node in order for a split to be attempted.

## Results

### Uncertainty and sensitivity analysis

**Figure 1:**
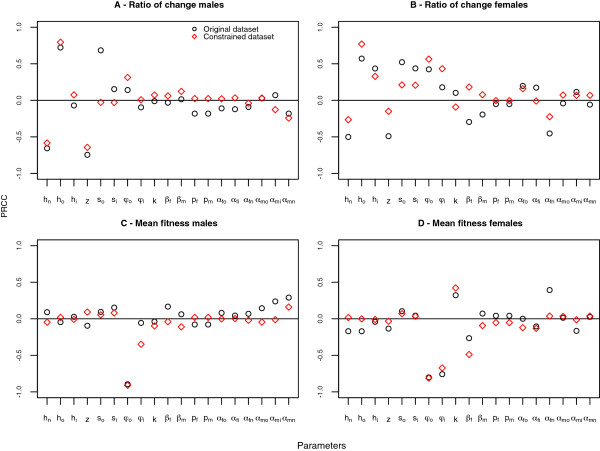
**Plot of Partial Rank Correlation Coefficients between each of the parameters in the model and the two outcome variables: mean fitness and ratio of change of resistance allele frequency, in both sexes (above zero increasing positive correlation, below zero increasing negative correlation. **The black circles refer to the coefficients calculated using the original dataset and the red diamonds coefficients were calculated using a dataset generated with the constrains: *α*_*fn *_< 0.2, *s*_*o *_> 0.5, *φ*_*i *_< 0.8, *s*_*i *_< 0.5, derived from the classification trees analysis. The parameters symbols in the x-axis are defined in Table 2.

LHS was used to generate a dataset for sensitivity analysis. The procedure was first replicated 10 times so that the model predictive precision could be assessed. The standard errors (se) and coefficient of variation (cv) of the outcome variables between replications are small (se: 0.001-0.3; cv: 0-0.005), suggesting that the predictive precision of the model does not depend on the LHS generated dataset. Statistic evidence (t-test, p-value < 0.05 in all replications) indicates a difference between sexes on both fitness and rate of change of resistance. Parameter sensitivity was performed to quantify how a change in an input parameter value causes a change in the outcome variables. Partial rank correlation coefficients calculated between each of the input parameters and the outcome variables are shown in Figure 1, in black circles in all panels. This analysis allows to assess the relative importance of the parameters in driving resistance and how it affects fitness, especially the magnitude of the correlation with the synergist, *k*.

The rate of spread of resistance (Figure 1, A and B)) is most sensitive to parameters values of selection and dominance coefficients and fitness cost (*s, h, z*). The correlation is negative in niches where insecticide is not employed and positive when it is present. In males (A), dominance and selection coefficients inside the house (*h*_*i*_,*s*_*i*_) have little effect on the ratio of change, presumably because only a small fraction is exposed.

The negative correlation between mean fitness and baseline fitness levels penalties (*φ*_*o*_ and *φ*_*i*_) is the strongest of all in both sexes. Male (C) and female (D) mean fitness are also sensitive to the parameters *α*: male PRCC coefficients are positive with mean fitness in all niches and females PRCC coefficients are positive in the insecticide free niche and negative in the other two.

Overall, changes in parameter *k* do not appear to have a big impact in the mean fitness of the population. In females the parameter *k* is positively correlated but small in magnitude and *β*_*f*_ (the proportion that meet both the synergist and insecticide) shows also only a small negative correlation. Both *k* and *β*_*f*_ show no correlation with mean fitness in the male population.

### Allele frequency ratio under two extreme scenarios of synergist effectiveness

**Figure 2:**
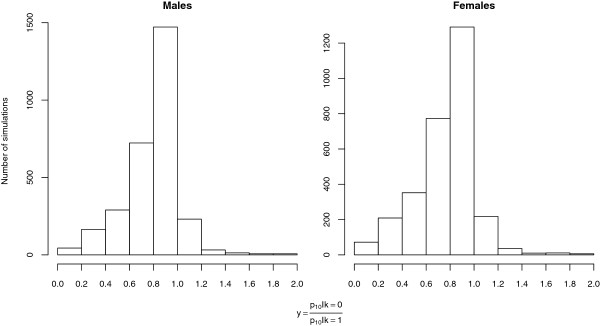
**Histogram of the ratio of resistance allele frequency at generation 10 in the extremes cases of a fully effective (*k *= 0) compared to an inefficient (*k *= 1) synergist:**$y=\frac{p_{10}|k=0}{p_{10}|k=1}$). Only values of *y *< 2 shown, which constitute 99.3% of the number of simulations. Values higher than 1 (*y *> 1) indicate the counter-intuitive result, i.e., that the synergist presence drives resistance faster.

**Figure 3:**
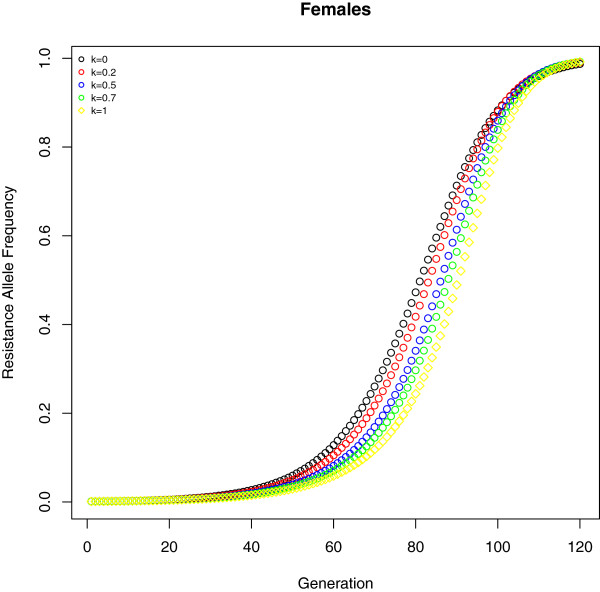
**How synergists affect the spread of resistance: Predicted resistance allele frequency in females for different values of synergist impact (*k *= 0,0.2,0.5,0.7,1) in a specific setting (*h*_*n *_= 0.37,*h*_*o *_= 0.50,*h*_*i *_= 0.07,*z *= 0.87,*s*_*o *_= 0.47,*s*_*i *_= 0.46,*φ*_*o *_= 0.11,*φ*_*i *_= 0.33,*β*_*m *_= 0.02,*β*_*f *_= 0.82,*α*_*fo *_= 0.41,*α*_*fi *_= 0.50,*α*_*fn *_= 0.10,*α*_*mo *_= 0.38,*α*_*mi *_= 0.34,*α*_*mn *_= 0**.***28*) that scored*y > 1***.

Figure 2 shows the rate change of allele frequency comparing the extremes cases of a fully effective (*k *= 0) and of a inefficient (*k *= 1) synergist. In most cases (90%) *y* is smaller than 1, which is intuitively the most likely outcome, i.e., resistance spreads slower in the presence of the synergist. Effect of synergist in males and females is not strictly comparable but is overall similar. Most importantly, Figure 2 shows that the difference between the two scenarios is small, most of the ratios are between 0.8 and 1, implying that the delay in the spread of resistance caused by the synergist is not very large. Nevertheless, what was not expected was that in approximately 10% of the cases the rate of allele spread can be higher when the synergist is fully effective (*y *> 1). Figure 3 shows the predicted frequency of the resistance allele under different values of k (ranging from 0 to 1) in a scenario which *y *> 1 to illustrate the difference in the spread of resistance. As an example, at generation 70 the predicted frequency when the synergist is inefficient (*k *= 1) is 0.11 and when is fully effective (*k *= 0) is 0.26.

### Logistic regression

Results of the logistic regression are presented in Table 4. Each one unit of increase in the parameter value in question will increase/decrease ( + /− signal of the estimate) the log odds of the unexpected event (*y *> 1), the odds are the exponentiated values of the estimates (*OR *=* e*^*Estimate*^), shown also in Table 4. The parameters *β*_*f*_, *β*_*m*_ and *α*_*mi*_ are not significative (p-values > 0.05) and appear to have no impact on the outcome. An increase in the parameters *s*_*o*_, *h*_*o*_, and *φ*_*o*_ increases considerably the odds off the counter-intuitive result (*y *> 1) and a increase of *α*_*fn*_, *h*_*i*_, *s*_*i*_, *φ*_*i*_, *α*_*fi*_, *h*_*n*_ and *z* slightly decreases the odds. Parameters related to the niche where insecticide is not deployed (*n*) do not have much impact on the counter-intuitive result, which appears to be governed mainly by the values of the parameters in the niche where insecticide is being applied for other reasons outside the house (*o*) and the niche insecticide inside the house (*i*).

**Table 4:** Logistic regression: parameter coefficient estimates, standard error, p-value and odds ratio

	**Estimate**	**Std. Error**	**p-value**	***OR***
Intercept	7.614	0.975	< 0.05	2.026e+03
*h*_*n*_	-3.215	0.553	< 0.05	4.016e-02
*h*_*o*_	7.828	0.685	< 0.05	2.511e+03
*h*_*i*_	-8.761	0.696	< 0.05	1.567e-04
*z*	-3.057	0.515	< 0.05	4.703e-02
*s*_*o*_	8.109	0.688	< 0.05	3.324e+03
*s*_*i*_	-7.645	0.681	< 0.05	4.785e-04
*φ*_*o*_	3.797	0.416	< 0.05	4.455e+01
*φ*_*i*_	-6.929	0.544	<0.05	9.794e-04
*β*_*f*_	0.190	0.387	0.6	1.209e+00
*β*_*m*_	0.378	1.833	0.8	1.460e+00
*α*_*fi*_	-6.584	0.753	<0.05	1.383e-03
*α*_*fn*_	-22.538	1.447	<0.05	1.628e-10
*α*_*mi*_	-2.205	3.016	0.46	1.103e-01
*α*_*mn*_	-0.948	0.472	0.04	3.875e-01

Additionally, to compare these two niches, a series of Wilcoxon signed-rank tests were performed. From the results of the tests it was determined that all the parameters in the niche where insecticide was encountered outside the house (*α*_*mo*_, *α*_*fo*_, *φ*_*o*_, *s*_*o*_ and *h*_*o*_) were significantly higher than the equivalent parameters in the niche inside the house in the simulations that led to the unexpected outcome.

### Classification trees

**Figure 4:**
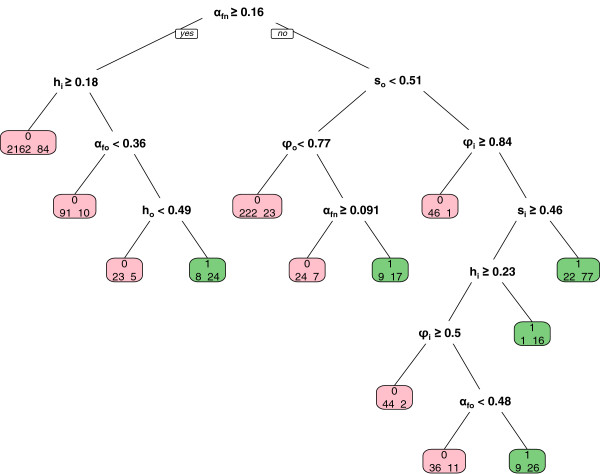
**An example of a classification tree of outcomes ***y *> 1 **(class 1) and ***y *< 1 **(class 0) using all the parameters in the model except *k*. **The items displayed in the nodes in the tree diagram are: the criterion for making the decision (e.g.: *α*_*fn *_≥ 0.1626), the predicted class for that terminal node (0 or 1) and the number of observations correctly classified to the class versus the number misclassified, achieved by cross-validation (e.g.: 2162/84). To proceed through the diagram at a given node move to the left branch if the stated condition is true (yes) and to the right if false (no).

The classification tree in Figure 4 is a tree pruned to avoid overfitting the data, that minimizes the cross-validated error [28]. The parameters actually selected by the algorithm (shown to have discriminant value) to construct the tree shown were: *h*_*i*_,* h*_*o*_,* s*_*i*_,* s*_*o*_,*α*_*fn*_,* α*_*fo*_,* φ*_*i*_ and *φ*_*o*_. The proportion of observations correctly classified at each leaf can be used to represent the likely proportion of similarly classified observations of unsampled data at the field conditions defined by that terminal node [29]. The proportions of classifications on the five terminal nodes that predicted the class 1 ranged from 0.059 ( $\frac{1}{(1+16}$) to 0.35 ( $\frac{9}{9+17}$). Further simulated datasets, with higher number of observations, produced slightly different trees, but they agree on the parameters selected for their construction and have the same basic structure.

The parameters most closely associated with the counter intuitive results are consistent between logistic regression and classification trees. Logistic regression is considered to be a more potent method, [30] but the schematic nature of the trees provides a clearer understanding of the interactions between the parameters and does offer a set of rules to follow in order to try and achieve a particular outcome.

### Constrained datasets

Objective analysis using logistic regression and classification trees led to a subjective conclusion as to why counter-intuitive results (*y *> 1) occur (see later). To validate the results new datasets with constrains on the parameters based on the tree decision criteria (e.g.: *α*_*fn *_< 0.2, *s*_*o *_> 0.5, *φ*_*i *_< 0.8, *s*_*i *_< 0.5), were generated with the expectation of reducing substantially the number of observations where *y *> 1. From 3,000 simulations produced with the previous constrain none resulted in *y *> 1. To end the analysis, a new dataset was generated with the above constrains to examine the impact of the different parameters on mean fitness when all observations lead to *y *< 1. Figure 1 shows in red the PRCC results calculated with this constrained dataset. The estimates values for the original dataset (black circles) and the constrained dataset (red diamonds) are similar, which implies that the presence of the counter-intuitive scenarios where *y *> 1 did not influence the overall correlation between the parameters and mean fitness. There was, however, a difference in the PRCC between the parameter *s*_*o*_(selection in the environment with insecticide outside the household) and the ratio of change of resistance allele frequency in the male population, it shows no correlation in this dataset while it did in the original dataset. The most plausible explanation is that male selective pressure occurred mostly in the niche with insecticide employed outside. This selection coefficients were reasonable high in the simulations that led to the unexpected outcome, so it can be considered normal that it showed impact in the original dataset and not in the new dataset.

### Dynamics of spread

The dynamics of spread was investigated by checking allele frequency at generation 1000 and determining resistance status (allele fixed if frequency greater than 0.99, lost if smaller than 0.001 and at equilibrium if change of frequency smaller than 0.001 in the last 50 generations) in the original dataset, subset with simulations that scored *y *> 1 and constrained dataset. Results are shown in Table 5.

**Table 5:** Dynamics of spread of resistance allele frequency

	**% Loss**	**% Fixation**	**% Equilibrium**
Original Dataset	49	30	21
Original Subset with y >1	3	72	25
Constrained dataset	8	79	13

Percentage of simulations that lead to loss, fixation and equilibrium of resistance allele in the original dataset, subset of the original dataset with simulations that scored *y *> 1 and constrained dataset based on the classification tree criteria (*α*_*fn *_< 0.2, *s*_*o *_> 0.5, *φ*_*i *_< 0.8, *s*_*i *_< 0.5).

## Discussion

Protection against vector borne diseases predominantly depends upon the usage of insecticides. Different strategies of delivery, in combination or independently, can be enforced while trying to minimise the emergence or impact of resistance. This study presents a model where mosquitos face an heterogeneous environment of four different niches, considering the use of insecticides outside the household and the existence of insecticide free areas (refugia). The model also allows to check the effect on resistance spread of new generation long-lasting insecticidal nets, that incorporate a synergist, reported to have improved increased efficacy against pyrethroid-resistant malaria vectors [15,31]. It would be simple to add more niches, essentially adding extra columns to Table 1, which is a major benefit of developing such a flexible methodology. For example, the outside niche could have high or low levels of insecticide. Under these conditions of different insecticide concentrations the resistance allele may be recessive or dominant respectively, with a huge impact on rate of resistance [20]. It would also be possible to allow for mosquitoes to be exposed to more than one niche, by multiplying the fitness in each niche. This would however increase the complexity of the model and, as presented, the model demonstrates how interesting results can be derived from simple approaches.

Here it was only considered the existence of different niches with different use of single insecticides, not allowing for deployment of a second insecticide with a different mode of action. This would require a new model assuming 2 loci, each encoding resistance to a single insecticide. These 2 locus models are simple in principle [32] but in the present model of multiple niches it would increase the number of parameters significantly (total of 9 genotypes for each sex and increase the number of potential niches to reflect different combinations of insecticides) and would not be beneficial in the current exploration of the effect of the synergist.

The calibration of the model with field data proved to be problematic, hence the decision to sweep a range of values and check the outcomes. The restriction on male proportion inside the house, less than 20%, was set based on personal communication since the numbers of males that enter households is rarely reported in hut trials, consequently it must be seen as a rough estimate. This work, in spite of the simplicity of the model, is illustrative of the advantages of modelling; an overall understanding that would not be possible by specific calibration and also the emergence of non-intuitive outcomes. Mathematical models have been used to expose other so non-intuitive results such that indoor residual spray (IRS) of insecticides in conjunction with bed nets can show antagonism, arising via interference of their modes of action while it is mostly assumed that the two tools have synergistic benefits in reducing malaria transmission [33].

Experimental studies have been conducted in the field to assess the potential of prototypes of bed nets that incorporate a pyrethroid insecticide in the side panels and the synergist PBO plus insecticide on the roof. An experimental hut trial in Tanzania was not able to demonstrate an improvement in efficiency (measured mortality, passage through holes and feeding rates) when compared to standard insecticide impregnated nets against *Culex quinquefasciatus*[31] and only moderate performance against pyrethroid-resistant *A. gambiae* M taxon was reported in another trial in southern Benin [34]. However, Killeen and colleagues [35] noted that the manufacturer of the prototype claims only that the net has greater efficacy than its predecessor and that their simulations corroborates this claim, which the findings of this work also support.

From the PRCC analysis is straightforward to infer that the success of control campaigns depends mostly on the proportion of mosquitoes that encounters insecticides and on the fitness scaling factors *φ*, that define survival of SS genotypes when meeting insecticides. These last parameters were introduced to emphasise possible differences among niches in the impact on fully susceptible SS genotypes when facing insecticides. Unsurprisingly the control measures are more effective the higher their values. The inclusion of this parameter was crucial because it considers the complexity of fitness, and incorporates the differential environmental effects of insecticides across different genotypes.

The present model includes two parameters that relate to the effects of application of a synergist in combination with a insecticide, *k*, the reduce survival due the synergist and the proportions of mosquitoes, *β* (males and females), that meet both chemicals. The magnitude of the correlation between males mean fitness and *k* and *β*_*m*_ is very small, presumably due to the small proportion of males that is exposed to insecticide in an household, but *k* and *β* only correlates moderately with females mean fitness and ratio of change of allele frequency. This indicates that the synergist has a small impact in controlling the population, but even small values of *k* will help to recover the effect of the insecticide, and possibly this is the main contribution of the synergist. Nevertheless adding synergists to bed nets does decrease the rate at which resistance spreads in about 90% of scenarios.

It was not possible to use the model to investigate the overall impact of changes in fitness in the mosquito population dynamics, which would require a more elaborate model incorporating demography (e.g. [36]), and more specifically to investigate the effects of targeting mainly females, decreasing their fitness more than that of males which are generally regarded as determining overall population regulation. The finding that a situation can arise in which having a fully effective synergist in place contributes to intensify the spread of resistance is the most interesting result of this work. The setting in which it emerges is very specific, it encompasses a strong selective pressure for resistance in the niche outside the household (mean *s*_*o *_= 0.62 while mean *s*_*i *_= 0.41; these and the following figures come from the simulated subset where *y *> 1), most mosquitoes are exposed to insecticides (mean *α*_*n *_for females is 0.16 and 0.44 for males) and there are different dominance values in the niches were insecticide is deployed (mean *h*_*o *_= 0.61 and mean *h*_*i *_= 0.37).

In these circumstances the insecticide in the bed nets will be largely ineffective and the pressure for selection is weak. It seems as if this niche is acting as a refugia for susceptibles, that will contribute with their susceptibles genes for the next generation, therefore decreasing the resistance allele frequency. If a fully effective synergist (*k *= 0) is present, the fitness of all genotypes inside the house will be zero (*k* affects the 3 genotypes equally, so all mosquitoes die irrespective of their genotype) and the next generation will be mostly composed by progeny of survivors from the niche outside the household where selection for resistance was high. An hypothesis is that in this particular case the synergist is removing the refugia of weak selection in the house thereby magnifying the effects of selection for resistance outside the house.

Males and females showed the same patterns of spread, 49% converged to fixation, 30% to loss and 21% to equilibrium. Equilibrium in single niche models (such as the single equation approach used in [20]) can only be achieved if there is heterozygote advantage [37] which was not postulated in the model because dominance coefficient *h* lie between 0 and 1 (Table 3). The balancing effects of different selection and dominance acting in different environments in the same population seems to be able to keep the resistance allele frequency at equilibrium. As an hypothetical example, a mutation which is dominant for insecticide resistance but has a large, recessive effect on fitness. As resistance starts to spread, most R alleles are in heterozygotes which resist insecticides and do not pay the fitness penalty. As R increases in frequency the proportion of RR homozygotes increases, so fitness penalties escalate until an equilibrium occurs. In effect, the marginal fitnesses (i.e. the average over all niches) generated by Table 1 and Equations 1 to 7 result in the heterozygote being the most fit in some simulations. As far as it was possible to verify it has not been reported in the field, possibly because it is not currently regarded as a likely occurrence.

The three dynamics of spread were predicted in subsequent analysis (Table 5) by estimating allele frequency for 1,000 generations. In the simulations that scored *y *> 1, 3% eventually lead to loss, 72% to fixation and 25% to equilibrium. It is overall a worse picture than with the original dataset. The choice of parameter values is important, for example constraining the dataset reduces the possibilities of reaching equilibrium, i.e., fixation of resistance was much more likely. Analysing only the simulations that lead to fixation in the original dataset generated results very similar (not shown) to Figure 1 and Table 4.

These results emphasise a very important fact often overlooked in modelling resistance: that it is highly dangerous to consider selection in only a single niche, isolated from other selection pressures, and then extrapolate the results from the single niche to the whole population. In this case it seems reasonable to conclude that adding effective synergists will reduce selection for resistance in the household niche because all three genotypes are killed. The impact that a fully effective synergist will have in disease transmission is a fundamental question, that cannot be directly answered by the results presented here, because it is not clear how the genetic concept of fitness translates into demographic factors such as mosquito population size and longevity that determine the intensity of disease transmission. On the other hand, as note above, if synergist throws most of the selection pressure onto another niche then overall the rate of selection for resistance may increase. Consequently the public use of insecticide within the home (predominantly as wall sprays and/or bed nets) cannot be investigates isolated from other insecticide applications that mosquitoes may encounter during their lifetime. This suggest that the malaria community is correct in being alarmed at the often uncontrolled use of insecticides in applications such as agriculture [38,39].

## Competing interests

The authors declare that they have no competing interests.

## Authors’ contributions

IMH and SB developed the model. SB implemented the model, performed the sensitivity analysis, carried out the simulations and wrote the manuscript. IMH reviewed the manuscript. Both authors read and approved the final manuscript.

## References

[B1] WHO World Malaria Report: 2011 World Health Organization 2011 1 259

[B2] Murray CJL Rosenfeld LC Lim SS Andrews KG Foreman KJ Haring D Fullman N Naghavi M Lozano R Lopez AD Global malaria mortality between 1980 and 2010: a systematic analysis Lancet 2012 379 413 31 10.1016/S0140-6736(12)60034-8 22305225

[B3] Nauen R Insecticide resistance in disease vectors of public health importance Pest Manag Sci 2007 63 628 633 10.1002/ps.1406 17533649

[B4] Labbé P Alout H Djogbénou L Pasteur N Weill M Evolution of Resistance to Insecticide in Disease Vectors 2011 Massachusetts, USA: Elsevier

[B5] Committee on Strategies for the Management of Pesticide Resistant Pest Populations NRC Pesticide Resistance: Strategies and Tactics for Management 1986 Washington: The National Academies Press

[B6] Chitnis N Smith T Steketee R A mathematical model for the dynamics of malaria in mosquitoes feeding on a heterogeneous host population J Biol Dyn 2008 2 259 285 10.1080/17513750701769857 22876869

[B7] Koella JC Lynch PA Thomas MB Read AF Towards evolution-proof malaria control with insecticides Evol Appl 2009 2 469 480 10.1111/j.1752-4571.2009.00072.x PMC3352447 25567892

[B8] Read A Lynch P Thomas M How to make evolution-proof insecticides for malaria control PLoS Biol 2009 7 e1000058 19355786 10.1371/journal.pbio.1000058 PMC3279047

[B9] Gourley SA Liu R Wu J Slowing the evolution of insecticide resistance in mosquitoes: a mathematical model Proc R Soc A 2011 467 2127 2148 10.1098/rspa.2010.0413

[B10] Hermsen R Hwa T Sources and Sinks: A Stochastic Model of Evolution in Heterogeneous Environments Phys Rev Lett 2010 105 24 248104 21231560 10.1103/PhysRevLett.105.248104 PMC4038430

[B11] Metcalf RL Mode of action of insecticide synergists Annu Rev Entomol 1967 12 229 256 10.1146/annurev.en.12.010167.001305 5340719

[B12] B-Bernard C Philogène BJR Insecticide synergists: role, importance, and perspectives J Toxicol Environ Health A 1993 38 199 223 10.1080/15287399309531712 8433403

[B13] Guillet P N’Guessan R Darriet F Traore-Lamizana M Chandre F Combined pyrethroid and carbamate ’two-in-one’ treated mosquito nets: field efficacy against pyrethroid-resistant Anopheles gambiae and Culex quinquefasciatus Med Vet Entomol 2001 15 105 112 10.1046/j.1365-2915.2001.00288.x 11297094

[B14] Oxborough RM Mosha FW Matowo J Mndeme R Feston E Hemingway J Rowland M Mosquitoes and bednets: testing the spatial positioning of insecticide on nets and the rationale behind combination insecticide treatments Ann Trop Med Parasitol 2008 102 717 727 10.1179/136485908X337553 19000389

[B15] Vestergaard-Frandsen PermaNetⓇ3.0-Features [ http://www.vestergaard-frandsen.com/permanet/permanet-3/features]

[B16] Roush RT Tabashnik BE Pesticide Resistance in Arthropods London: Chapman and Hall 1990: 303.

[B17] McKenzie J Batterham P Predicting insecticide resistance: mutagenesis, selection and response Philoso Trans R Soc London. Ser B: Biol Sci 1998 353 1729 1734 10.1098/rstb.1998.0325 PMC1692398 10021773

[B18] Mallet J The evolution of insecticide resistance: Have the insects won? Trends Ecol Evolut 1989 4 336 340 10.1016/0169-5347(89)90088-8 21227375

[B19] Bourguet D Genissel A Raymond M Insecticide resistance and dominance levels J Econ Entomol 2000 93 1588 1595 10.1603/0022-0493-93.6.1588 11142285

[B20] Barbosa S Black WC Hastings I Challenges in estimating insecticide selection pressures from mosquito field data PLoS Negl Trop Dis 2011 5 e1387 10.1371/journal.pntd.0001387 22069506 PMC3206009

[B21] Bourguet D Prout M Raymond M Dominance of insecticide resistance presents a plastic response Genetics 1996 143 407 416 8722792 10.1093/genetics/143.1.407 PMC1207273

[B22] Zhivotovsky LA Feldman MW Bergman A Fitness patterns and phenotypic plasticity in a spatially heterogeneous environment Genet Res 1996 68 241 248 10.1017/S0016672300034212 9062081

[B23] Kidwell JF Clegg MT Stewart FM Prout T Regions of stable equilibria for models of differential selection in the two sexes under random mating Genetics 1977 85 171 183 838269 10.1093/genetics/85.1.171 PMC1213615

[B24] Sanchez M Blower S Uncertainty and sensitivity snalysis of the basic reproductive rate: tuberculosis as an example Am J Epidemiol 1997 145 1127 1137 10.1093/oxfordjournals.aje.a009076 9199543

[B25] Gu W Novak RJ Predicting the impact of insecticide-treated bed nets on malaria transmission: the devil is in the detail Malar J 2009 8 256 10.1186/1475-2875-8-256 19917119 PMC2780451

[B26] R Development Core Team R: A Language and Environment for Statistical Computing 2010 [ http://www.R-project.org]

[B27] Breiman L Classification and Regression Trees Belmont (Calif.): Wadsworth International Group 1984: 358.

[B28] Therneau T Atkinson EJ An introduction to recursive partitioning using the RPART routines 2011 1 67 [ http://r.789695.n4.nabble.com/attachment/3209029/0/zed.pdf]

[B29] Miller J Franklin J Modeling the distribution of four vegetation alliances using generalized linear models and classification trees with spatial dependence Ecol Model 2002 157 227 247 10.1016/S0304-3800(02)00196-5

[B30] Austin PC A comparison of regression trees, logistic regression, generalized additive models, and multivariate adaptive regression splines for predicting AMI mortality Stat Med 2007 26 2937 2957 10.1002/sim.2770 17186501

[B31] Tungu P Magesa S Maxwell C Malima R Masue D Sudi W Myamba J Pigeon O Rowland M Evaluation of PermaNet 3.0 a deltamethrin-PBO combination net against *Anopheles gambiae* and pyrethroid resistant *Culex quinquefasciatus* mosquitoes: an experimental hut trial in Tanzania Malar J 2010 9 21 10.1186/1475-2875-9-21 20085631 PMC2817703

[B32] Curtis C Theoretical models of the use of insecticide mixtures for the management of resistance Bull Entomol Res 1985 75 259 265 10.1017/S0007485300014346

[B33] Yakob L Dunning R Yan G Indoor residual spray and insecticide-treated bednets for malaria control: theoretical synergisms and antagonisms J R Soc Interface 2010 8 799 806 21084340 10.1098/rsif.2010.0537 PMC3104351

[B34] N’guessan R Asidi A Boko P Odjo A Akogbeto M Pigeon O Rowland M An experimental hut evaluation of PermaNet(Ⓡ) 3.0, a deltamethrin-piperonyl butoxide combination net, against pyrethroid-resistant *Anopheles gambiae* and *Culex quinquefasciatus* mosquitoes in southern Benin Trans R Soc Trop Med Hyg 2010 104 758 765 10.1016/j.trstmh.2010.08.008 20956008

[B35] Killeen G Okumu F N’guessan R Coosemans M Adeogun A Awolola S Etang J Dabiré R Corbel V The importance of considering community-level effects when selecting insecticidal malaria vector products Parasit Vectors 2011 4 160 160 10.1186/1756-3305-4-160 21838903 PMC3189155

[B36] White MT Griffin JT Churcher TS Ferguson NM Basáñez MG Ghani AC Modelling the impact of vector control interventions on *Anopheles gambiae* population dynamics Parasit Vectors 2011 4 153 10.1186/1756-3305-4-153 21798055 PMC3158753

[B37] Hartl DL Clark AG Principles of population genetics Sinauer Associates 2007 652

[B38] Mouchet J Agriculture and Vector Resistance Insect Sci Appl 1988 9 1 6

[B39] N’Guessan R Darriet F Guillet P Carnevale P Traore-Lamizana M Corbel V Koffi AA Chandre F Resistance to carbosulfan in *Anopheles gambiae* from Ivory Coast, based on reduced sensitivity of acetylcholinesterase Med Vet Entomol 2003 17 19 25 10.1046/j.1365-2915.2003.00406.x 12680920

